# Assisted Extraction of Hemp Oil and Its Application to Design Functional Gluten-Free Bakery Foods

**DOI:** 10.3390/molecules30122665

**Published:** 2025-06-19

**Authors:** Noemi Baldino, Mario F. O. Paleologo, Mariateresa Chiodo, Olga Mileti, Francesca R. Lupi, Domenico Gabriele

**Affiliations:** Department of Information, Modeling, Electronics and System Engineering (D.I.M.E.S.), University of Calabria, Via P. Bucci, Cubo 39C, I-87036 Rende, CS, Italy; oraldo.paleologo@unical.it (M.F.O.P.); mary.chiodo99@gmail.com (M.C.); o.mileti@dimes.unical.it (O.M.); francesca.lupi@unical.it (F.R.L.); domenico.gabriele@unical.it (D.G.)

**Keywords:** batter, cupcake, microwaves, rheology, ultrasonication

## Abstract

*Cannabis sativa* L. is known for its high-value compounds, like Cannabidiol (CBD) and Cannabidiolic Acid (CBDA). It is widely used in the pharmaceutical and food industries. Different extraction methods, like Soxhlet and maceration, are commonly employed to obtain its extracts. High temperature and long extraction time can influence the yield and the purity of the extracts, affecting the quality of the final product. This study focused on optimizing CBD oil extraction from hemp inflorescences and its incorporation into a gluten-free bakery product for functionalization. Dynamic maceration (DME), assisted by ultrasound and microwave irradiation, was used. Our study explored the impact of varying sonication times (three distinct durations) and microwave powers (three levels, applied for two different irradiation times) on the resulting extracts. HPLC analysis was performed on these extracts. Subsequently, we used hemp flour and hemp oil to bake gluten-free cupcakes, which were fortified with the extracted CBD oil. Rheological characterization was used to investigate the cupcake properties, along with stereoscopic, color and puncture analysis performed on the baked samples. The most effective extraction parameters identified were 30 s of microwave irradiation at 700 W, yielding 45.2 ± 2.0 g of CBD extract, and 15 min of sonication, which resulted in 53.2 ± 2.5 g. Subsequent rheological characterization indicated that the product exhibited mechanical properties and a temperature profile comparable to a benchmark, evidenced by a height of 4.1 ± 0.2 cm and a hardness of 1.9 ± 0.2 N. These promising values demonstrate that hemp oil and hemp flour are viable ingredients for traditional cakes and desserts, notably contributing increased nutritional value through the CBD-enriched hemp oil and the beneficial profile of hemp flour.

## 1. Introduction

*Cannabis sativa* L. (family *Cannabaceae*) is a widespread crop species cultivated for various industrial uses, such as textile, constructions, bioplastics, pharmaceuticals, and food [1,2]. In the textile industry, hemp recently gained attention due to the increasing demand for natural fibers. When blended with cotton fibers, hemp can improve fabric breathability and thermal regulation [3]. In construction, hemp is used to produce hempcrete, a biocomposite constituted of hemp shives, lime, and water, which is beneficial due to its ability to sequester CO_2_ from the atmosphere [3]. Hempcrete is also used in 3D-printing applications [2,3]. Short hemp fibers are employed in wood plastic composites to recycle polypropylene from textile waste [2,3]. Moreover, hemp biomass is suitable for thermochemical conversion in biochar via pyrolysis [2]. In the food industry, hemp is gaining prominence due to its high content of protein, fiber, and unsaturated fatty acids [4,5]. Hemp seeds are rich in edestin and albumin, which are easily digestible and are used as functional protein in food processing [4,5]. Hemp proteins are also used in stabilizing emulsions for functional foods, cosmetics, and pharmaceuticals [5]. Hemp milk is emerging as a plant-based dairy alternative to animal-derived ones [2,5], while hemp flour is used to enrich protein content and improve the texture of gluten-free breads [4,5]. Beyond its traditional uses, hemp yields a wide array of biomolecules recognized for their significant functional and nutritional value [6]. *Cannabis sativa* L. is usually classified according to its Δ⁹-tetrahydrocannabinol (THC) content. Particularly speaking, if the THC is above 0.3%, the plant is for drug use; if it is below 0.3%, the plant can be used for industrial purposes [6,7].

The inflorescences of the hemp plant are rich in various bioactive compounds, such as cannabinoids, terpenes, and flavonoids. These compounds are of significant interest for numerous pharmaceutical, cosmetic, and nutraceutical applications, as previously mentioned [8]. Among cannabinoids, Cannabidiol (CBD) and its acid precursor, Cannabidiolic Acid (CBDA), are the most extensively studied due to their promising therapeutic applications. CBD, in particular, is recognized for its anti-inflammatory, anxiolytic, neuroprotective, and antiepileptic properties [9]. Consequently, the research aims to improve the CBD extraction process. To maximize the CBD yield, the inflorescences require decarboxylation. This can involve a heating process that converts CBDA into CBD [6,9].

During hemp processing, a considerable amount of waste is invariably generated, often comprising 30% to 50% of the total mass. This includes stems, certain inflorescences, powders, and residual fibers post-oil extraction [6]. The waste is still rich in oil, waxes, and bioactive compounds. The extraction of oils, waxes, and cannabinoids from hemp by-products can be carried out through different methodologies. Each technique is based on specific physicochemical principles, and has advantages and limitations in terms of yield, purity of extracts, and sustainability of the process [10]. Recent studies [6,9] have shown that Soxhlet allows for high yields of cannabinoids, although the high extraction temperature may result in degradation of some thermolabile compounds. The solvent demands and long processing times, however, limit the sustainability of this technique.

CBD and CBDA can also be extracted by dynamic maceration using ethanol, which was found in literature to be the best in terms of CBD yield obtained from the hemp matrix [9,11]. Moreover, supercritical carbon dioxide extraction is one of the most advanced and sustainable techniques for processing hemp and acts as a selective non-polar solvent for lipophilic compounds. Using the optimal pressure and temperature parameters, the chemical profile of the extracts can be modulated, resulting in cannabinoids, terpenes, and waxes with high purity [12]. Recent studies have shown that ultrasound-assisted extraction is particularly effective in preserving thermolabile compounds, such as CBDA, and in obtaining extracts with high concentrations of cannabinoids and terpenes [8,13]. Microwave-Assisted Extraction (MAE) is a promising technique for the extraction of bioactive compounds from *Cannabis sativa* [14]. This method enables rapid and uniform heating, which increases intracellular pressure and causes cell wall rupture, facilitating the release of cannabinoids, terpenes, and flavonoids [15]. Compared to traditional techniques, MAE offers significant advantages, such as reduced extraction time, lower solvent consumption, and increased yield and selectivity, especially for thermolabile compounds such as CBDA, due to precise temperature control. Recent studies have shown that combining ethanol as a solvent with microwave energy enables the rapid production of cannabinoid-rich extracts [14,16].

Hemp is considered a functional ingredient with an excellent nutritional profile, given the high levels of polyunsaturated fatty acids (EFAs) and essential amino acids (EAAs) contained in hemp seeds [17]. The use of hemp flour, protein, and oil in food is a current and growing challenge. In recent years, scientific research has focused on the use of hemp flours for the preparation of baked goods and confectionery products, including gluten-free [12,18]. The use of hemp flour, therefore, is still limited, and the few products on the market are poorly known. Hemp flour, compared to wheat flour, has a different protein profile. Hemp flour is low in starch and unable to form a gluten network. While this absence of gluten is problematic for individuals with celiac disease, it is beneficial for dough formation [19]. The deficiency of gluten affects the texture [20]. Particularly in the cake industry, the use of the proper ingredients is critical to achieving the desired texture. For cupcakes, oils and fats are key components in obtaining the desired texture, as they help to stabilize air bubbles during creaming processes, interact with starches and proteins, and contribute to the emulsifying effect [21]. In this work, the replacement of hemp oil enriched with CBD oil and hemp flour for the preparation of cupcakes is considered, because of the large amount of predominantly saturated fat that this type of cake requires [21,22].

Given the preceding context, this research pursued two primary objectives. First, it aimed to optimize the extraction of CBD oil from *Cannabis sativa* inflorescences by evaluating the enhanced yield from dynamic ethanol maceration when assisted by microwave and ultrasound techniques, since, although the process is studied in literature, comparative studies are lacking, especially with and without a decarboxylation step. The decarboxylation step is crucial to convert non-psychoactive cannabinoid acids (like THCA and CBDA) into their active, psychoactive forms (THC and CBD). Second, the study focuses on utilizing the antioxidant-rich lipid phase to enrich the hemp oil and develop gluten-free cupcakes prepared with hemp flour, thereby trying to yield a food product of significant nutritional impact.

## 2. Results and Discussion

This section presents the extraction yields derived from different techniques, employing both as-is and decarboxylated inflorescences, utilizing microwave and ultrasound-assisted extraction. We report the quantification of CBD and CBDA in the extracts, followed by the rheological characterization of baked products formulated with hemp oil. The physical characteristics of the finished baked goods are then presented.

### 2.1. Extraction

All the dynamic maceration extractions (DMEs) were conducted on inflorescences sourced from the same hemp plant. The same DMEs were performed on both unprocessed (as-is) inflorescences and decarboxylated inflorescences, with and without pretreatments with ultrasound and microwaves to improve the CDB extraction. The extracted quantity is reported as milligrams of oil per gram of raw material loaded (mg/g_rm_). The extraction, performed without assisted methods but under the same conditions as the dynamic maceration with pretreatments, gives a quantity of 186 ± 11 mg/g_rm_ using HI and 120 ± 12 mg/g_rm_ for DHI. Table 1 shows the yields obtained by ultrasonic-assisted dynamic extraction on the inflorescences, without decarboxylation (HI) and after decarboxylation (DHI).

The data in Table 1 reveal that extraction yield in oil from HI and DHI samples increases with longer sonication times, confirming the positive influence of sonication on the extraction process.

For the HI sample, a slight increase in extraction yield occurs between 5 and 10 min of treatment, after which the yield appears to plateau at 15 min. This trend suggests that sonication enhances the release of lipid compounds from the inflorescences. The high-frequency sound waves likely generate pressure waves and cavitation within the plant matrix, effectively breaking down cell walls and facilitating lipid release [8,9].

Decarboxylated inflorescences (DHIs) showed a similar trend but with slightly lower initial extraction yields at 5 min. This could be attributed to the structural modification of acidic cannabinoids (e.g., CBDA) during decarboxylation, potentially reducing their availability or extractability by sonication [9]. Nevertheless, an increase in the yield with time and a similar trend of the HI are also observed for the DHI samples extraction [8]. In general, the extraction yield obtained for HI samples is higher than that obtained with DHI samples at all the investigated times. The temperature employed to facilitate decarboxylation and the conversion to CBD likely results in a deterioration effect on the hemp sample [23]. The combined effect of dynamic extraction and sonication increases the extraction capacity compared with using sonication side by side with traditional maceration extraction [23].

Table 2 shows the results obtained from dynamic extraction conducted under assisted conditions using the microwave technique for 30 s and 60 s.

The analysis of the data at different microwave powers shows how the applied power and duration affect the efficiency of the extraction process. In particular, the use of higher powers leads to higher extraction yields for both matrices. This trend shows that increasing microwave power promotes the extraction of desired compounds. In fact, microwaves generate rapid and uniform heating of the plant matrix, inducing molecular vibrations that promote the breakdown of plant cells and the release of soluble compounds, such as cannabinoids and essential oils [8]. The higher the power applied, the more intense the interaction of the microwaves with the matrix, increasing the efficiency of the extraction process. At both the 30 s and 60 s treatments, decarboxylated inflorescences consistently yielded less than those from dynamic maceration alone. This suggests a degradation effect from the heat-induced decarboxylation, which ultimately impacts the total extract yield. It appears that microwaves, even at intermediate power, are more efficient than maceration, especially in the treatment of non-decarboxylated inflorescences. This suggests that the use of microwaves significantly improves extraction compared to traditional methods, due to the rapid heating and dielectric effect that facilitates heat transfer and solubilization of target compounds [13].

Extraction from hemp inflorescences with microwaves for 60 s has significantly higher yields than using sonication and maceration without pretreatment.

This can be, again, attributed to the physical principles related to the use of microwaves, which generate rapid and uniform agitation of molecules within the plant matrix, creating a thermal and mechanical effect [8,23]. Microwaves induce an increase in temperature and pressure within the plant cell, which promotes the breakdown of cell walls and facilitates the release of soluble compounds. The energy provided by microwaves is therefore more effective than sonication, which relies mainly on acoustic waves to produce pressure waves that generate a cavitation effect, which is less direct in extracting soluble substances [16].

The analysis of yield data from dynamic, ultrasound-assisted, and microwave-assisted extraction reveals that the ultrasound technique is the most efficient, achieving the highest extraction yields. This result agrees with the literature where it is found that for the extraction of pectin from grapefruits, comparing microwave and sonication techniques, the highest extraction yields are observed with sonication [24].

### 2.2. Extracts Analysis by HPLC

The extracts derived from inflorescences subjected to different treatment methodologies were analyzed by HPLC to determine their CBD and CBDA content. Table 3 shows the results obtained from the analysis of the extracts obtained from the inflorescences after sonication treatment for different times.

Also, in terms of cannabinoids, as with the yields evaluated in the previous section, the amounts obtained using sonication are higher than those obtained by dynamic maceration alone. As sonication times increase, the amounts of CBD and CBDA extracted increase. It is confirmed that the use of decarboxylated inflorescences leads to maximizing the amounts of CBD obtained as a result of CBDA conversion. The results obtained agree with what has also been found in the literature [8].

Table 4 shows the results obtained by pretreating the inflorescences with microwaves.

As indicated by the results in Table 4, there is a correlation between increased microwave power and longer treatment times and the resulting higher CBD and CBDA content in the extracts. This again confirms that the CBD yield rises while the CBDA yield diminishes due to decarboxylation. In contrast, the extraction yield at the minimum power level is similar to that achieved by dynamic maceration of the as-is inflorescences. Using 30 s as the microwave treatment time is satisfactory, and the CBD value obtained at all powers investigated is comparable to that obtained with 60 s. A comparison of the techniques employed reveals that, with the decarboxylated matrix, higher CBD yields are achieved through sonication compared to the microwave treatment. This finding, however, differs from a prior study on hemp inflorescences which, when comparing dynamic maceration, ultrasonic, and microwave techniques, identified microwave extraction as yielding optimal CBD results [9].

### 2.3. Rheological Results

Cupcake batter formulations were investigated. A standard formulation was initially prepared to assess its rheological properties and baking performance. Subsequently, alternative formulations were developed where butter was replaced by hemp oil (functionalized with 2% *w*/*w* CBD oil for antioxidant activity) and wheat flour by hemp flour.

All resulting batters underwent rheological analysis to confirm their suitability for cupcake batter. The initial formulation, based on butter and type 00 flour and following a traditional Italian recipe, was prepared and analyzed. Subsequently, butter was replaced with CBD-rich hemp oil. Finally, type 00 flour was substituted with increasing ratios of maize starch and hemp flour, ultimately leading to a complete replacement with hemp flour.

**Figure 1:**
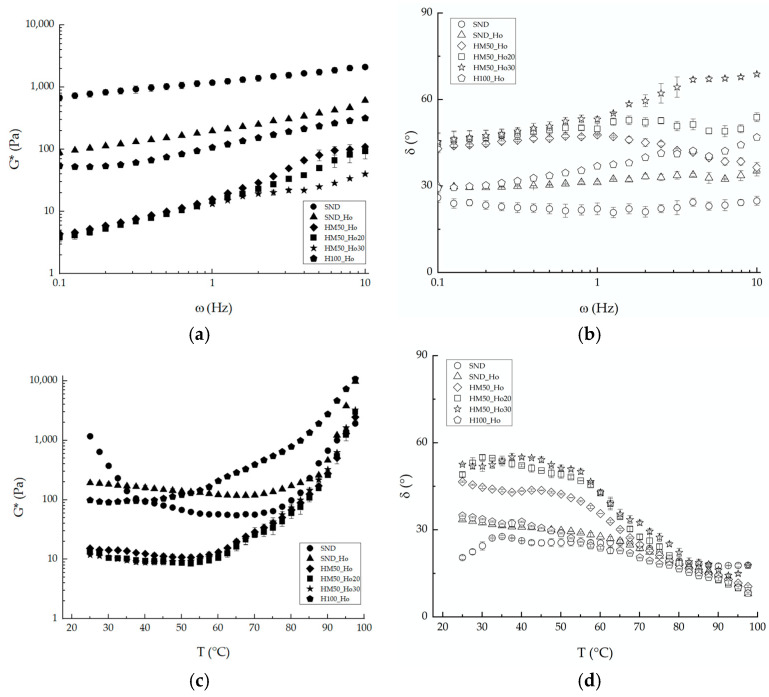
Frequency sweep test at 25 °C (**a**,**b**) and time-cure tests (**c**,**d**) for all the samples investigated, in terms of complex modulus, G* (**a**,**c**), and phase angle, δ (**b**,**d**). SND refers to the standard sample; SND_Ho is the formulation standard with hemp oil (Ho); HM50_Ho is the formulation with hemp flour (H) and maize starch (M); HM50_Ho20 is the formulation HM50_Ho with hemp oil reduction of 20%; HM50_Ho30 is the formulation HM50_Ho with hemp oil reduction of 30%; H100_Ho is the formulation with hemp flour (H) and hemp oil (Ho).

The results of frequency sweep tests on the studied samples at a temperature of 25 °C are reported in Figure 1. The graph clearly indicates that the reference sample (SND) exhibits the most consistent texture, characterized by higher G* values, low frequency dependence, and the lowest phase angle values, consistently around 20°. Replacing butter with hemp oil (SND_Ho) resulted in a slight decrease in batter consistency and a reduction in the texture of the mixture. The 00 flour in the SND_Ho batter was replaced with the mixture of hemp flour and maize starch, and the frequency analysis shows that the obtained batter had a significantly lower consistency. Then, because of the low consistency, the amount of oil was reduced to 20% (SND_Ho20) and subsequently to 30% (SND_Ho30) without affecting the rheological behavior of the batter. The phase angle trend follows that of G*, then a decrease in the complex modulus corresponds to a more liquid and less structured behavior, with higher phase angle values. With the purpose of improving the structure, the hemp/maize mixture was replaced with 100% of hemp flour (H100_Ho). The latter sample showed a consistency comparable to that obtained with the SND_Ho sample.

The frequency sweep tests, particularly the trend of G* with changing frequency, were analyzed using the weak gel rheological model, and the resulting characteristic parameters are presented in Table 5. Consistent with the time-cure trend, these parameters indicate that dough strength tends to decrease as temperature increases. This behavior was observed for the samples without hemp flour: SND and SND_Ho. In contrast, doughs prepared with hemp flour exhibited a slight increase in strength at 60 °C, potentially due to the early structuring effect of the sample. Notably, the sample composed of 100% hemp flour and no starch (H100_Ho) showed a sharp increase in this parameter at 60 °C.

The SND_Ho sample exhibits similar characteristics to the SND sample, except for the decrease in modulus between 25 and 40 °C due to the substitution of butter with hemp oil. Samples with hemp flour and starch (HM50_Ho, HM50_Ho20, and HM50_Ho30) are all at the same consistency values and show an increase in modulus from 60 °C up to 100 °C. The HM100_Ho sample shows initial consistency comparable to that of the reference standards. In particular, the sample prepared with hemp flour and no starch (H100_Ho) achieves a sharp increase in this parameter at 60 °C.

The study of G* and delta as temperature is changed (time-cure) allows us to better visualize all these effects observed at the three different frequency temperatures. In particular, high G* values are observed for the SND sample at 25 °C due to the presence of butter, in agreement with the literature [25]. Up to 80 °C, the values remain constant, and then increase markedly in the 80–100 °C range. The increase in consistency starts around 40 °C and ends at 100 °C with values very similar to those achieved by the SND_Ho batter. The high initial consistency of the sample ensures that the batter retains the incorporated air, leading to a soft and porous final texture [18,26].

Due to its lack of starch, hemp flour exhibits poor structuring capability at elevated temperatures. This inherent property makes it difficult for gluten-free doughs to develop a well-structured form when hemp flour is used [20]. The absence of starch in the hemp matrix is generally balanced by adding starch or proteins and polysaccharides capable of imparting the desired structure [4]. The use of oil instead of butter resulted in a batter with a softer consistency near 40 °C. This results in good workability of the dough without losing the structure and consistency that allow the dough to incorporate air for the development of the final aerated structure [21]. The H100_Ho sample’s texture and protein content, derived from both eggs and flour, enabled the matrix to structure to values higher than the standard, yet this is still optimal.

### 2.4. Baking Results

**Figure 2:**
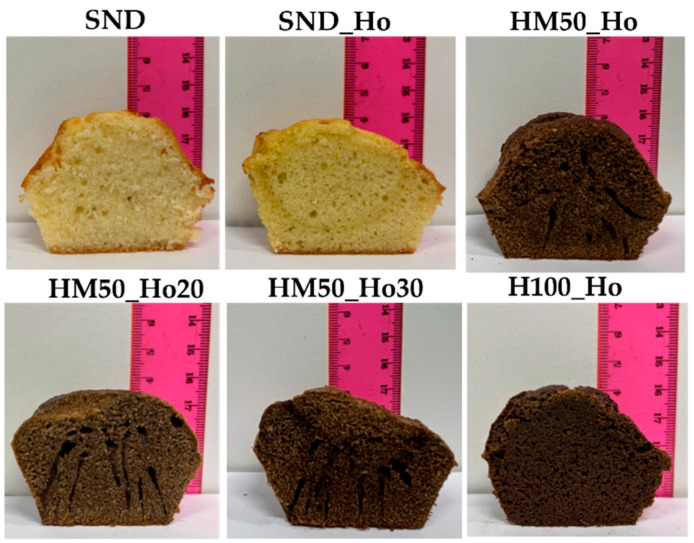
Section of cooked samples showing the appearance, the crumb, and the heights of cupcakes. SND is the standard sample; SND_Ho is the formulation standard with hemp oil (Ho); HM50_Ho is the formulation with hemp flour (H) and maize starch (M); HM50_Ho20 is the formulation HM50_Ho with hemp oil reduction of 20%; HM50_Ho30 is the formulation HM50_Ho with hemp oil reduction of 30%; H100_Ho is the formulation with hemp flour (H) and hemp oil (Ho).

All the samples studied were baked to evaluate the characteristics of the finished product. Figure 2 shows images of the cooked samples and shows how the variation in ingredients used leads to different internal stratifications. It is observed that the low consistency of HM50_Ho, HM50_Ho20, and HM50_Ho30 samples leads to the formation of internal channels induced by the low batter structuring, which do not allow for a good retention of air in the matrix during baking and consequently the development of a homogeneous internal structure. In contrast, sample HM100_Ho shows a homogeneous, compact and uniform texture similar to the SND sample. Figure 2 visually confirms the rheological finding of low consistency in samples HM50_Ho, HM50_Ho20, and HM50_Ho30 until the structuring temperature. This low consistency promotes the formation of non-homogeneity.

The heights of the samples were also evaluated (see Table 6), confirming the uniform growth of sample H100_Ho with height values comparable to those obtained with the standard batter. The results are in agreement with the rheological analysis that shows the H100_Ho samples most similar to the standard one. The ability to achieve a product height comparable to the standard is a highly significant parameter. This is primarily due to the common leavening challenges encountered with most gluten-free formulations, which frequently result in products exhibiting a deficit in vertical development relative to standard equivalents.

**Figure 3:**
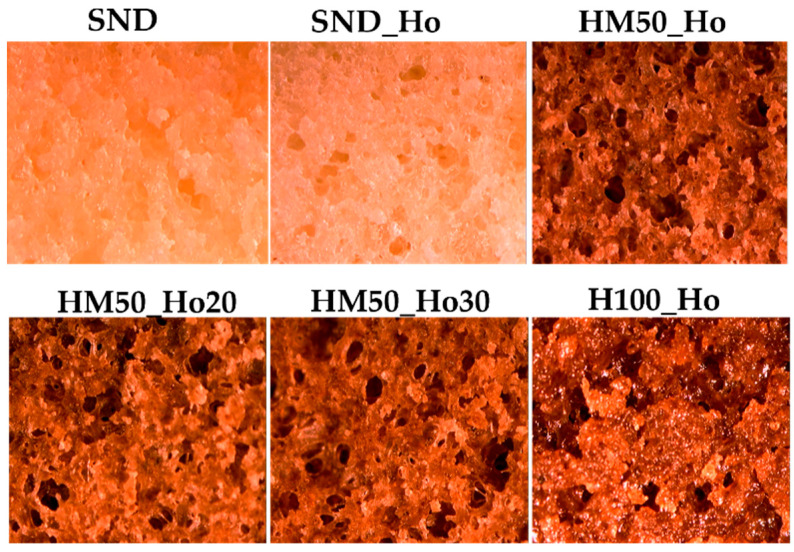
Stereoscopic images for all samples for the crumb section of cupcakes. SND is the standard sample; SND_Ho is the formulation standard with hemp oil (Ho); HM50_Ho is the formulation with hemp flour (H) and maize starch (M); HM50_Ho20 is the formulation HM50_Ho with hemp oil reduction of 20%; HM50_Ho30 is the formulation HM50_Ho with hemp oil reduction of 30%; H100_Ho is the formulation with hemp flour (H) and hemp oil (Ho).

The sample observation by stereoscopic analysis (see Figure 3) shows that sample H100_Ho has high homogeneity, comparable to that of the SND and SND_Ho samples. It is also observed that the other samples show more pronounced holes, suggesting the formation of channels, as observed in Figure 2.

Compression tests (puncture tests) were finally performed on the samples to evaluate the texture of post-baking samples. In fact, the typical porous texture provides softness [26], which can be related to the texture of the material. The slope of the stress–strain line relative to the first linear section corresponds to the elasticity index (E_I_). The parameters evaluated for the samples are shown in Table 7.

The results indicate that incorporating hemp oil as a substitute for butter reduces the elasticity index (E_I_) of the samples made with type “00” flour. Furthermore, replacing wheat flour with a 1:1 mixture of hemp flour and starch appears to have no significant impact on the EI. Notably, a decrease in the amount of hemp oil used correlates with a reduction in softness. The analysis of the hardness parameter also confirms this trend, showing that SND, SND_Ho and H100_Ho samples result as those with higher hardness. The hardness parameter is evaluated to estimate the strength required for the first chew of the product [27]. Samples with maize starch result in more crumbles compared to the other samples. Hardness data are in accordance with work in the literature for gluten-free bakery food [28].

L*, a*, and b* parameters were measured to identify the color of the cupcakes, inside (crumb) and on the surface (crust), and the results are shown in Table 8. Substituting butter for hemp oil does not significantly alter the color parameters, as also observed from the images in Figure 2. In contrast, substituting flour for hemp flour significantly alters both parameter values and appearance as seen from Figure 2. In particular, the addition of hemp oil decreases the L* value, as in the literature with the substitution of palm oil with sunflower oil [21]. Furthermore, the addition of hemp flour causes a decrease in the L* parameter due to the browning induced by hemp flour, as well as an increase in the parameter a* indicative of a greater tendency to reddish values of the dough. Also b* decreases although less markedly. Between crust and crumb, it is observed that the addition of hemp flour results in a homogeneity of values for the parameters L* and b*, while the parameter a* tends in all cases to be higher for the crust part and lower for the crumb part, as also found in the literature [29].

These results are corroborated in the literature [18,30], where hemp flour is shown to significantly reduce the L* parameter and increase the red and yellow tendency of the crust and crumb compared to the standard sample.

## 3. Materials and Methods

### 3.1. Materials

Flowers were manually divided from the hemp plant (*Cannabis sativa* L.) to obtain hemp inflorescences. The plant was supplied by Le Querce S.r.l (Montalto Uffugo, Italy), and the experimental cultivation is Futura 75 (carried out at Spezzano Sila, Cosenza, Italy), certified with D9-THC content below 0.3% (*w*/*w*). Hemp protein flour (HPF) was kindly provided by Eco Officina Agraria S.r.l. (Arezzo, Italy) and is composed of fats 24% *w*/*w*, carbohydrates 9% *w*/*w*, proteins 57% *w*/*w*, and moisture 10% *w*/*w*. The hemp flour particle size is 58.05 ± 0.23 μm.

For HPLC analysis, acetonitrile, formic acid and pure water were supplied by Carlo Erba Reagents (Val-de-Reuil, France).

CO_2_ (purity > 99.99%) was supplied by SIAD Spa (Bergamo, Italy) for supercritical extraction.

CBD and CBDA standards were supplied by Sigma-Aldrich Co. (St. Louis, MO, USA).

Butter (Despar, Casalecchio di Reno, Italy), hemp oil (Soc. Agricola Antichi Grani, Narni, Italy) with the addition of 2% *w*/*w* of HI extract, eggs (Delizie del sole, San Martino Buon Albergo, Italy), saccarose (Dolciando, San Martino B.A., Italy), 00 wheat flour (Tre mulini, Bologna, Italy), UTH whole milk (Land, Castenedolo, Italy), maize starch (Tre mulini, Bologna, Italy), and baking powder (Dolciando) were used to obtain the cupcakes. For 00 wheat flour, the particle size is 26.6 ± 0.8 μm.

### 3.2. Extraction and Extracts Characterization

#### 3.2.1. Extraction

According to literature [6], the dynamic maceration extraction (DME) was performed using 1.0 ± 0.1 g of plant material and 40 mL of ethanol. The extraction process consisted of three successive steps. In the first step, the sample was suspended in the solvent and stirred on a magnetic stirrer for 15 min. At the end of this phase, the exhausted plant matrix was recovered and resuspended in an additional 40 mL of ethanol for another 15 min under the same stirring conditions (second step). Finally, the residue was subjected to a third extraction step by agitation in 20 mL of ethanol for a further 15 min. The resulting solutions from all three steps were combined to obtain a final extract volume of 100 mL.

We performed extractions using both raw hemp inflorescences (HIs) and decarboxylated hemp inflorescences (DHIs). The decarboxylation process involved heating the inflorescences at 140 °C for 30 min in a ventilated oven (FD 53, BINDER GmbH, Tuttlingen, Germany), a method consistent with the published literature [6,31].

To enhance the extraction yield of cannabinoids, two integrated extraction techniques were evaluated, in which the first step of the dynamic maceration process was assisted by ultrasound and microwave treatments, respectively.

In the first case, 1.0 ± 0.1 g of hemp inflorescences was suspended in 40 mL of ethanol and subjected to sonication using a SONOREX SUPER RK 100/RK 100 H ultrasonic bath (BANDELIN, Berlin, Germany). Three sonication times were tested: 5, 10, and 15 min. Following sonication, the standard DME protocol was resumed starting from the second extraction step, as described above.

In the second case, the first step of dynamic maceration was assisted by microwave irradiation. Specifically, 1 ± 0.1 g of hemp inflorescences or stems was suspended in 40 mL of ethanol and put inside a microwave oven (OK OMW 2022 W, 20 L, 700 W). Extractions were conducted at three different power levels: high (700 W), medium (540 W), and low (120 W), and for two exposure time intervals, respectively: 30 and 60 s. For hemp stems, the microwave-assisted step was further optimized, and the most effective pretreatment time was applied. As with the ultrasound-assisted method, the subsequent two steps of DME were carried out according to the standard protocol.

The extracts obtained from the dynamic maceration processes, including standard DME as well as ultrasound- and microwave-assisted extractions, were separated from the solvent using a Rotavapor system (Heidolph G3, Hei-VAP Value, Heidolph Instruments, Wood Dale, IL, USA) operated at 80 °C and equipped with a vacuum pump (Edwards, Manor Royal, Crawley, West Sussex) to improve the efficiency of solvent removal. This procedure was applied to all experimental trials. At the end of the process, solvent-free hemp extracts were obtained.

All extraction tests were performed in triplicate. Once all extracts were obtained, the quantitative yield of the different extraction processes was calculated using the following Equation (1):

(1) $$yield=\frac{mass\;extract\;(mg)}{feed\;mass\;(g)}$$

#### 3.2.2. HPLC Analysis

Extracts were analyzed by a Smartline HPLC system (Knauer, Berlin, Germany) to evaluate the CBD and CBDA content. The instrument consists of a degasser, a pump, and a UV detector 2600. A 150 mm and 2 mm i.d. C18 Ascentis, with a precolumn (Supelco, Darmstadt, Germany) was used. The column temperature was set at 32 °C. The mobile phase was composed of 0.1% *v*/*v* formic acid in acetonitrile (A) and 0.1% *v*/*v* formic acid in water (B) at a flow rate of 0.1 mL/min. An elution gradient was imposed as follows: 0–13 min, 60% A; 13–17 min, from 60 to 80% A; 17–22 min, from 80% to 90% A, which was maintained for eight minutes, and finally, a 15 min post-running time was imposed. This protocol agrees with the literature [6,9]. Absorbance spectra were recorded every 1 s, between 200 and 450 nm, with a bandwidth of 8 nm. Chromatograms were acquired at 210, 220, 235, and 275 nm [9,31]. All tests were performed in triplicate. The calibration curves were obtained for both CBD and CBDA at concentrations of 0.01, 0.025, 0.1, 0.5, and 1 mg/mL. Before injection into the HPLC system, the extracts were filtered using a 0.45 µm PTFE filter.

**Figure 4:**
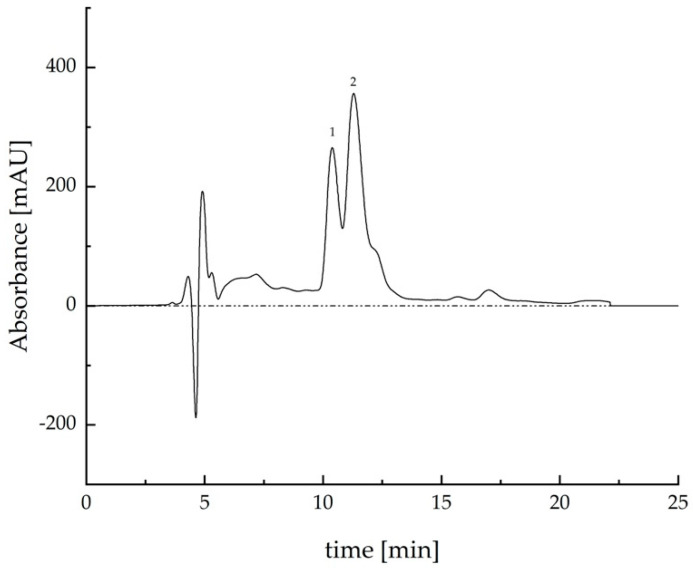
Chromatogram of HI extract using the sonication technique for 15 min, in terms of absorbance at varying times; 1 is the pick related to CBDA and 2 is related to CBD.

An example of the chromatogram is reported in Figure 4, in which two pick characteristics can be observed for hemp inflorescences extracts: the CBDA pick (1) and the CBD (2) pick. From the literature, the CBDA is earlier than the CBD [9].

### 3.3. Cupcake Preparation

The formulations were shown in Table 9 and were developed from a traditional Italian cupcake (SND) recipe.

For each formulation, 500 g of batter was prepared. The ingredients were mixed using a planetary mixer (KitchenAid, Benton Harbor, MI, USA) following three steps: first sugar and butter (or hemp oil) are initially mixed at speed 6 for 2 min, then eggs and milk were added and mixed under the same conditions and finally, flour (00 flour or hemp flour with maize starch) was added and the whole mixture was mixed at the same speed for 3 min. Once the mixing stage is completed, the dough is put at 4 °C and allowed to rest for a minimum of 20 min.

For each formulation, the dough was prepared twice. No yeast was added to the dough samples prepared for the rheological tests to prevent bubble formation from interfering with the measurements.

For a qualitative evaluation of the cupcake characteristics, baking tests were performed. The doughs were prepared by also adding yeast, inserted after the last step of mixing, and stirring for an additional minute. Before baking, the dough was distributed into silicone cups (45 ± 1 g), which were then baked in a ventilated oven (Unox XF013, Stefania, Padua, Italy) at 160 °C for 20 min.

### 3.4. Rheological Dough Measurements

The doughs were characterized by rheological measurements carried out using a rotational rheometer (Anton Paar MCR 702e, Anton Paar AG, Graz, Austria) equipped with a parallel plate geometry (diameter = 20 mm; gap = 2 mm ± 0.1 mm) and a Peltier system for controlling the temperature. Frequency sweep tests were performed at 25 °C, 40 °C and 60 °C in a 0.1–10 Hz frequency range, in the linearity region, preliminarily evaluated by stress sweep and time sweep tests. Dynamic temperature ramp tests in the linear regime were also run, increasing the temperature from 20 to 100 °C, with a ramp rate of 1 °C/min. When running tests at high temperature, water loss was prevented by covering the sample rim with a thin layer of silicone oil (viscosity 1 Pa·s, VWR Chemicals, Fontenay-sous-Bois, France).

To ensure the repeatability of the data, each sample was prepared twice, and each test was run three times on every sample. All presented data are expressed as mean values with their standard deviations. The data can be reported in terms of complex modulus, G*, obtained as follows:

(2) $$G^{*}=\sqrt{\left({G^{\prime }}^{2}+{G^{\prime \prime }}^{2}\right)}$$

where *G*′ is the storage modulus and *G″* is the loss modulus, while the phase angle, δ, is expressed as follows:

(3) $$\delta =\left(\frac{G^{\prime \prime }}{G^{\prime }}\right)$$

In rheological terms, the doughs behave like “weak gels” when subjected to frequencies between 0.1 and 100 Hz, which is the standard range for investigation. This material forms a three-dimensional network due to its interacting rheological units. The complex modulus of this network follows a power–law relationship**:**

(4) $$G^{*}=A\omega^{\frac{1}{z}}\;$$

where ω is the frequency, A is directly related to the strength of this three-dimensional network, and **z** provides insight into how much the network extends [32].

Data were reprocessed by software OriginPro (Version 2021b, OriginLab Corporation, Northampton, MA, USA). The same software was used to perform data fitting.

### 3.5. Cupcake Characterization

On the cooked samples, tests of color were performed using a colorimeter (Croma Meter CR-400, Konica Minolta, Tokyo, Japan), and the measurements were analyzed with the CieLab coordinates L*, the axis of lightness; a*, the axis of red/green transition; and b*, the axis of yellow/blue transition. A white standard plate with coordinates (L* = 97.02, a* = 0.14, b* = 2.26) was used for preliminary calibration.

Analyses were conducted on three samples, both on the outer surface (shell) and inside the sample.

Stereoscopic images were captured using a NIKON SMZ800 stereoscope (Magnification 10×, Tokyo, Japan) with SCOPE TEK DCM130 optical unit, equipped with OPTIKA Vision lite 2.0 reprocessing software. This allowed for the comparison and evaluation of the structure and size of the alveolus, which is typical for baked and leavened products such as a cupcake [33]. One measurement was performed on each prepared formulation.

**Figure 5:**
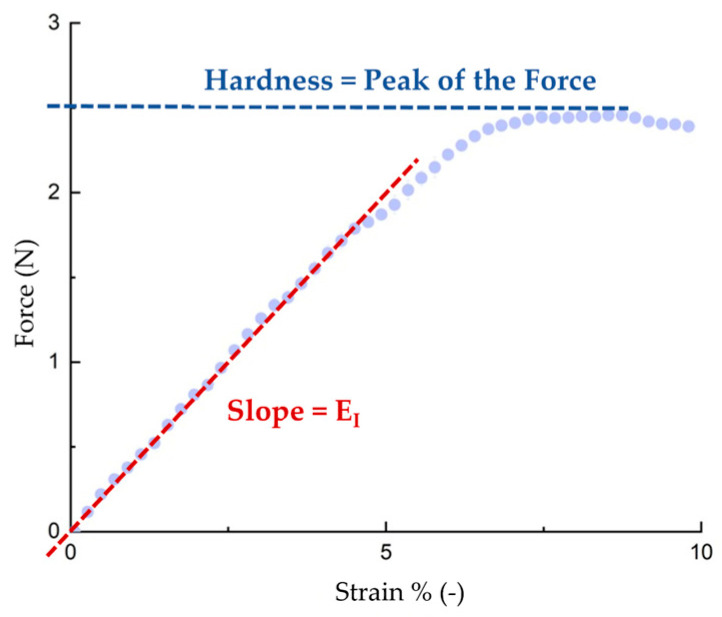
The typical force versus strain trend for a cupcake is presented. The trend for the SND sample is reported, and the key parameters are represented: hardness and the elasticity index (EI). Dotted line means the peak of the experimental points.

Puncture tests were performed using a tensile machine (Zwick-Roelle, ProLine Z005 TN, Ulm, Germany) equipped with a 5.0 kN load cell, to evaluate the mechanical resistance of baked samples in terms of elasticity index (E_I_). The elasticity index, on the other hand, can be calculated from the slope of the first linear section. The parameter provides information about the texture of the material. The hardness was quantified as the peak force recorded during the compression analysis, which can be interpreted as the force necessary for the initial bite [27,34]. An example graph for calculating the E_I_ and hardness parameters is shown in Figure 5. All tests were performed at room temperature on baked samples that were allowed to stand for one hour after baking [35]. The specimens were compressed with a piston of area 35.7 mm^2^ (diameter = 6.74 mm) at a constant rate of 2 mm/s [35].

### 3.6. Statistical Analysis

The results are reported as the means and standard deviation using software OriginPro (Version 2021b, OriginLab Corporation, Northampton, MA, USA).

## 4. Conclusions

This work focused on optimizing CBD-rich oil extraction from hemp inflorescences and explored incorporating hemp oil into gluten-free cupcakes to enhance their nutritional and functional properties, replacing traditional fats. The initial extraction step used dynamic maceration assisted by ultrasound and microwave irradiation. We analyzed both decarboxylated and non-decarboxylated inflorescences, investigating three sonication times and three microwave power levels across two irradiation times.

The results showed that increasing the sonication time led to higher extraction yields, particularly for non-decarboxylated inflorescences. While evident, the increase was less significant for decarboxylated samples, confirming the sonication’s general benefit to yield. Notably, a 10 min sonication time was sufficient to achieve the highest yield. Furthermore, microwave irradiation significantly improved extraction yields, especially for non-decarboxylated inflorescences, demonstrating a greater impact than sonication.

The HPLC analysis revealed that the highest CBD yield came from decarboxylated inflorescences, attributed to the decarboxylation process itself. The highest CBD yield was specifically achieved with the highest level of dynamic maceration assisted by microwaves, suggesting that microwaves not only facilitate cannabinoid diffusion but also enhance the decarboxylation reaction, thereby maximizing CBD extraction. Finally, the CBD-enriched oil was integrated into gluten-free cupcake recipes, substituting butter with the oil and 00 flour with hemp flour, aiming to create a functional gluten-free food product.

The rheological analysis revealed that replacing butter with CBD-enriched hemp oil reduced batter consistency. The subsequent, gradual incorporation of hemp flour led to a further decrease. However, when hemp flour was used exclusively, the consistency of the batter was restored to a level comparable to the traditional formulation, resulting in an optimal baked texture.

In conclusion, our findings demonstrate that dynamic maceration assisted by ultrasound and microwave irradiation is an efficient method for extracting CBD-rich oil. Furthermore, hemp oil presents a viable alternative to traditional oils and fats for creating functional foods.

## Figures and Tables

**Table 1:** Ultrasonic-assisted dynamic extraction yield (mg/g_rm_) for Hemp Inflorescences (HIs) and Decarboxylated Hemp Inflorescences (DHIs). The values of yield for HI and DHI are 186 ± 11 mg/g_rm_ and 120 ± 12 mg/g_rm,_ respectively.

Time of Sonication (min)	HI	DHI
5	171 ± 6	122 ± 1
10	262 ± 6	244 ± 5
15	276 ± 2	261 ± 2

**Table 2:** Yield (mg/g_rm_) of dynamic maceration assisted by 30 and 60 s microwave treatment for Hemp Inflorescences (HIs) and Decarboxylated Hemp Inflorescences (DHIs). The values of yield for HI and DHI are 186 ± 11 mg/g_rm_ and 120 ± 12 mg/g_rm,_ respectively.

Power, (W)	HI, 30 s	DHI, 30 s	HI, 60 s	DHI, 60 s
120	188 ± 13	142 ± 11	202 ± 7	152 ± 11
540	263 ± 9	243 ± 13	285 ± 27	273 ± 16
700	301 ± 2	282 ± 15	346 ± 10	304 ± 10

**Table 3:** Milligrams of Cannabidiol (CBD) and Cannabidiolic Acid (CBDA) extracts per g of raw load material (mg/g_rm_) for Hemp Inflorescences (HIs) and Decarboxylated Hemp Inflorescences (DHIs), after sonication treatment.

Time of Sonication (min)	CBD/CBDA	HI	DHI
0	CBD	13.78 ± 1.38	35.46 ± 0.30
	CBDA	26.28 ± 1.82	6.57 ± 0.03
5	CBD	15.19 ± 0.50	37.31 ± 1.41
	CBDA	28.19 ± 0.67	1.19 ± 0.06
10	CBD	19.46 ± 1.58	50.21 ± 3.17
	CBDA	33.32 ± 3.16	1.75 ± 0.16
15	CBD	22.74 ± 1.06	53.21 ± 2.78
	CBDA	37.51 ± 2.19	2.46 ± 0.23

**Table 4:** Milligrams of Cannabidiol (CBD) and Cannabidiolic Acid (CBDA) extracts per g of raw load material (mg/g_rm_) for Hemp Inflorescences (HIs) and Decarboxylated Hemp Inflorescences (DHIs), after microwave treatment for a time of 30 s and 60 s. The values of CBD and CBDA for HI are 13.78 ± 1.38 mg/g_rm_ and 26.28 ± 1.82 mg/g_rm_, while for DHI are 35.46 ± 0.30 mg/g_rm_ and 6.57 ± 0.03 mg/g_rm_, respectively.

Power (W)	CBD/CBDA	HI, 30 s	DHI, 30 s	HI, 60 s	DHI, 60 s
120	CBD	6.85 ± 0.07	23.30 ± 0.93	8.59 ± 0.77	25.17 ± 0.21
	CBDA	19.53 ± 1.03	1.39 ± 0.09	25.48 ± 1.78	1.32 ± 0.10
540	CBD	11.78 ± 0.54	38.10 ± 0.69	13.75 ± 0.46	41.27 ± 1.29
	CBDA	29.96 ± 1.36	1.48 ± 0.04	35.81 ± 2.80	2.78 ± 0.15
700	CBD	15.25 ± 0.84	45.29 ± 1.01	19.28 ± 0.98	48.21 ± 0.56
	CBDA	36.54 ± 2.29	2.51 ± 0.19	38.94 ± 0.90	5.64 ± 0.42

**Table 5:** Weak gel parameters A (strength) and z (structuration degree) for all investigated batter samples.

	25 °C		40 °C		60 °C	
Sample	A, Pa·s^1/z^	z, -	A, Pa·s^1/z^	z, -	A, Pa·s^1/z^	z, -
SND	1200 ± 130	4.30 ± 0.01	70 ± 3	3.70 ± 0.03	48 ± 4	4.10 ± 0.04
SND_Ho	200 ± 10	2.70 ± 0.01	150 ± 7	2.50 ± 0.01	110 ± 5	3.70 ± 0.02
HM50_Ho	20 ± 1	1.45 ± 0.02	13 ± 2	2.00 ± 0.01	20 ± 2	2.10 ± 0.01
HM50_Ho20	15 ± 2	1.50 ± 0.03	10 ± 1	2.10 ± 0.01	15 ± 1	3.10 ± 0.02
HM50_Ho30	12 ± 1	2.10 ± 0.01	11 ± 1	1.20 ± 0.01	20 ± 1	1.60 ± 0.01
H100_Ho	110 ± 3	2.40 ± 0.01	110 ± 5	2.35 ± 0.01	270 ± 15	3.20 ± 0.01

SND refers to the standard sample; SND_Ho is the formulation standard with hemp oil (Ho); HM50_Ho is the formulation with hemp flour (H) and maize starch (M); HM50_Ho20 is the formulation HM50_Ho with hemp oil reduction of 20%; HM50_Ho30 is the formulation HM50_Ho with hemp oil reduction of 30%; H100_Ho is the formulation with hemp flour (H) and hemp oil (Ho).

**Table 6:** Height values of the baked cupcake in millimeters (mm), reported as mean value ± standard deviation.

Sample	SND	SND_Ho	HM50_Ho	HM50_Ho20	HM50_Ho30	H100_Ho
Height	4.1 ± 0.1	3.9 ± 0.2	4.1 ± 0.1	3.6 ± 0.1	3.3 ± 0.1	4.1± 0.2

SND refers to the standard sample; SND_Ho is the formulation standard with hemp oil (Ho); HM50_Ho is the formulation with hemp flour (H) and maize starch (M); HM50_Ho20 is the formulation HM50_Ho with hemp oil reduction of 20%; HM50_Ho30 is the formulation HM50_Ho with hemp oil reduction of 30%; H100_Ho is the formulation with hemp flour (H) and hemp oil (Ho).

**Table 7:** Elasticity index (E_I_) and hardness, both in Newton (N), for baked cupcakes.

Sample	SND	SND_Ho	HM50_Ho	HM50_Ho20	HM50_Ho30	H100_Ho
E_I_	0.40 ± 0.05	0.20 ± 0.02	0.22 ± 0.02	0.18 ± 0.01	0.14 ± 0.01	0.19 ± 0.01
Hardness	2.75 ± 0.03	2.25 ± 0.03	1.80 ± 0.03	1.60 ± 0.03	1.50 ± 0.03	1.90 ± 0.01

SND is the standard sample; SND_Ho is the formulation standard with hemp oil (Ho); HM50_Ho is the formulation with hemp flour (H) and maize starch (M); HM50_Ho20 is the formulation HM50_Ho with hemp oil reduction of 20%; HM50_Ho30 is the formulation HM50_Ho with hemp oil reduction of 30%; H100_Ho is the formulation with hemp flour (H) and hemp oil (Ho).

**Table 8:** Color parameters in CieLab space, L*, a* and b*, of baked cupcakes.

	L*		a*		b*	
Sample	Crumb	Crust	Crumb	Crust	Crumb	Crust
SND	77 ± 1	56 ± 2	−3.2 ± 0.2	−1.4 ± 1.1	19 ± 1	35 ± 2
SND_Ho	79 ± 2	63 ± 2	−3.0 ± 0.3	−1.3 ± 0.8	23 ± 2	38 ± 1
HM50_Ho	29 ± 1	32 ± 1	2.7 ± 0.3	6.5 ± 0.8	17 ± 1	16 ± 1
HM50_Ho20	30 ± 1	32 ± 1	2.5 ± 0.2	7.3 ± 0.4	17 ± 1	15 ± 1
HM50_Ho30	33 ± 1	35 ± 1	2.7 ± 0.6	7.6 ± 0.3	16 ± 1	16 ± 1
H100_Ho	22 ± 1	25 ± 2	4 ± 0.4	8.3 ± 0.5	10 ± 2	12 ± 1

SND is the standard sample; SND_Ho is the formulation standard with hemp oil (Ho); HM50_Ho is the formulation with hemp flour (H) and maize starch (M); HM50_Ho20 is the formulation HM50_Ho with hemp oil reduction of 20%; HM50_Ho30 is the formulation HM50_Ho with hemp oil reduction of 30%; H100_Ho is the formulation with hemp flour (H) and hemp oil (Ho).

**Table 9:** Cupcake formulations. All the components were reported as weight percentage (%*w*/*w*).

Component	SND	SND_Ho	HM50_Ho20	HM50_Ho30	H100_Ho
Wheat flour (W)	22.5	22.5	0	0	0
Hemp flour (H)	0	0	11.25	11.25	22.5
Maize starch (M)	0	0	11.25	11.25	0
Sugar (S)	22.5	22.5	22.5	22.5	22.5
Eggs (E)	22.5	22.5	22.5	22.5	22.5
Hemp Oil (HO)	0	15.8	12.6	11.3	15.8
Butter (B)	22.5	0	0	0	0
Milk (M)	10	10	10	10	10
Baking powder (B)	0.5	0.5	0.5	0.5	0.5

SND is the standard sample; SND_Ho is the formulation standard with hemp oil (Ho); HM50_Ho is the formulation with hemp flour (H) and maize starch (M); HM50_Ho20 is the formulation HM50_Ho with hemp oil reduction of 20%; HM50_Ho30 is the formulation HM50_Ho with hemp oil reduction of 30%; H100_Ho is the formulation with hemp flour (H) and hemp oil (Ho).

## Data Availability

The data used to support the findings of this study can be made available by the corresponding author upon request.
